# Study protocol: Early neurological deterioration in patients with minor stroke, frequency, predictors, and outcomes in Vietnam single-centre study

**DOI:** 10.1371/journal.pone.0302822

**Published:** 2024-05-06

**Authors:** Dung Tien Nguyen, Ton Duy Mai, Phuong Viet Dao, Hung Tran Ha, Anh Tuan Le, Tuyet Trinh Thi Nguyen, Trung Xuan Vuong, Minh Cong Tran

**Affiliations:** 1 BachMai Stroke Center, BachMai Hospital, Hanoi, Vietnam; 2 VNU University of Medicine and Pharmacy, Hanoi, Vietnam; 3 Hanoi Medical University, Hanoi, Vietnam; 4 Department of Clinical Neuroscience, University of Oxford, Oxford, United Kingdom; Chinese Academy of Medical Sciences and Peking Union Medical College, CHINA

## Abstract

Early neurological deterioration (END) is progressive neurological deterioration with an increase in NIHSS score of 2 points or more in the first 72 hours from the onset of acute ischemic stroke. END increases the risk of poor clinical outcomes at day 90 of ischemic stroke. We will study the frequency, predictors, and outcomes of patients with END in a case-control study at a comprehensive stroke centre in Vietnam. of the design is a descriptive observational study, longitudinal follow-up of patients with minor stroke hospitalized at the Stroke Center of Bach Mai Hospital from December 1, 2023, to December 1, 2024. Minor stroke patients characterized by NIHSS score ≤ 5 hospitalized within 24 hours of symptom onset will be recruited. The estimated END rate is about 30%, relative accuracy ε = 0.11, 95% reliability, expected 5% of patients lost data or follow-up, and an estimated sample size of 779 patients. This study will help determine the END rate in patients with minor stroke and related factors, thereby building a prognostic model for END. Our study determined the END rate in patients with minor stroke in Vietnam and also proposed risk factors for minor stroke management and treatment.

## Introduction

Minor stroke is considered an acute ischemic stroke that has an NIHSS score ≤ 5 points, with the condition of a score of 0 on the cognitive section of the NIHSS scale [1–4]. Patients with minor stroke accounts for 30–50% of the general ischemic stroke population [5–8]. Although the initial clinical manifestations are mild neurological deficits, poor clinical outcomes rate (mRS 2–6) are for a very high rate of 23.5–38% [4, 9, 10]. Early neurological deterioration is related to reasonably expected poor clinical outcomes, accounting for 5–40% [11, 12]. Early neurological deterioration (END) in minor stroke is defined as a condition with progressive neurological deficit or an increase in NIHSS score of 2 points or more in the first 72 hours of illness [4, 11–14]. Therefore, identifying factors of minor stroke associated with END helps us offer appropriate treatment strategies to reduce the risk of poor clinical outcomes at day 90 [12].

In Vietnam, poor clinical outcome in minor stroke patients is related to initial NIHSS scores, clinical progression with NIHSS scores increasing above 4 in the first 24 hours, and intracranial artery stenosis [13]. However, the definition of END has not been made clear, and the minor stroke definition NIHSS ≤ 4 seems inappropriate. Outcomes, associated factors and frequency of minor stroke in Vietnam were not well characterised. There is a lack of research on minor stroke patients and also the END scale in Vietnam application leading to severe consequences in stroke management and treatment.

Therefore, in this study, we aim to determine the population of END subjects in a subgroup of minor stroke patients (NIHSS scores ≤ 5) hospitalized in the first 72 hours and determine factors (clinical and paraclinical characteristics, imaging, treatment) related to END based on regression analysis. This proposed case-control protocol for minor stroke is the first study conducted in Vietnam with 779 participants (a number estimated by statistical calculation).

## Methods

### Study design

**Fig 1:**
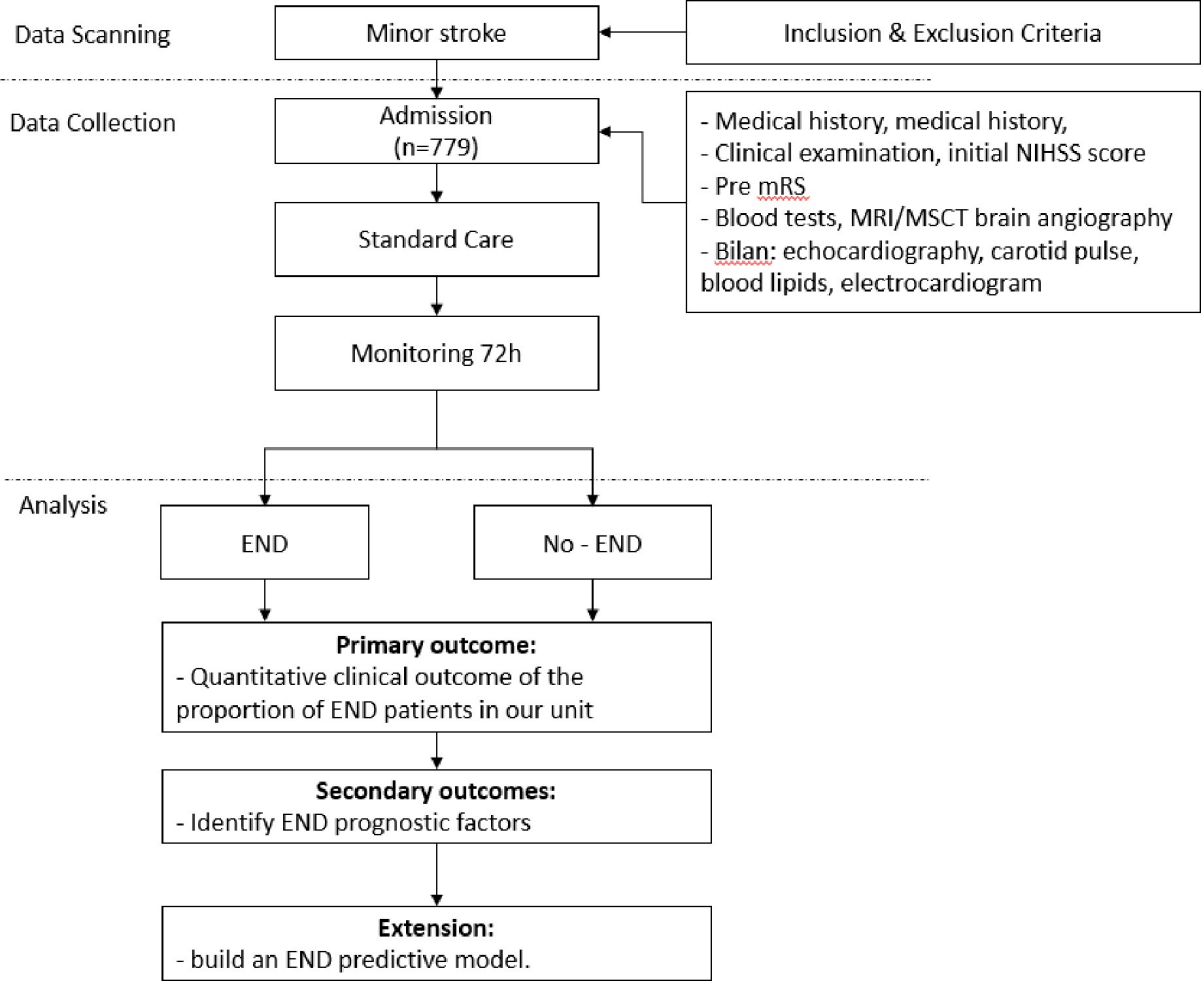
Flow chart of the study design. END is Early neurological deterioration; mRS is the Modified Rankin Score; and NIHSS is the National Institutes of Health Stroke Scale.

The study design is an observational, descriptive study, with longitudinal follow-up of all patients with minor stroke (NIHSS ≤ 5 points, with the condition of a score of 0 in the cognitive section of the NIHSS scale) hospitalized within 24 hours. It will be at the comprehensive stroke centre of Bach Mai Hospital from December 1, 2023, to December 1, 2024. A study flow chart is illustrated in Fig 1. Exclusion criteria for mRS before stroke ≥ 2 points; Pregnant and breast-feeding women; co-existence of other brain injury: traumatic brain injury, intracranial hemorrhage, subarachnoid hemorrhage, and brain tumour. Further information on the eligibility criteria for this study can be found in Table 1.

**Table 1:** Eligibility criteria for the study, with mRS, is the Modified Rankin Score, and NIHSS is the National Institutes of Health Stroke Scale.

**Inclusion criteria**
• Clinical diagnosis of stroke
• Aged > 18 years
• NIHSS ≤ 5 points, with the condition of a score of 0 in the cognitive section of the NIHSS scale
• hospitalized within 24 hours
• Informed consent
**Exclusion criteria**
• mRS before stroke ≥ 2 points
• Pregnant and lactating women
• Traumatic brain injury
• Brain haemorrhage
• Subarachnoid haemorrhage
• Brain tumour

### Ethical approval

All research documents were approved by the Ethics Committee of Bach Mai Hospital according to decision No. 4837/BM-HDDD. All patients and families agree to participate in the study and signed an informed consent. All concent data are obtained by both verbal and written format.

### Sample size calculation

We applied the sample size calculation formula to estimate a proportion, which in our study is the proportion of patients with END as the main criterion:

$$n=z_{1-\alpha }^{2}\frac{1-P}{\varepsilon^{2}P}$$

In which: n is the required research sample size; p, the proportion of patients with END; ε is an estimate of the allowable relative deviation between the rate obtained from the sample and the speed of the population; Z, the corresponding confidence coefficient, depends on the confidence limit (1-α) that the researcher chooses for their research; We took the value p = 0.3 according to the proportion of patients with END of 30% as research results by N. Boulenoir et al. in 2020, on the group of minor stroke NIHSS ≤ 5 points [15]. Choose relative accuracy, value d = 0.11. Reliability coefficient with α = 0.05. From there, we calculated the estimated sample size N = 741. With the expectation that 5% of patients would have missing or insufficient data, we took the expected sample size of 779 patients.

### Statistical analysis

We separated the data set of 779 subjects into two main groups, END and no-END. Demographic information, risk factors during stroke, NIHSS on admission, the etiology of stroke, and medications during hospitalization are compared between patients with and without END during the acute phase of stroke. All data inputs from the patients are recorded and reported in **Table 2**. The comparison is achieved by *t*-test (if normal distribution) or Kruskal-Wallis test (non-normal distribution) for continuous variables. The χ2 test is used to analyze categorical variables. Multivariate Logistic regression models after adjusting for potential confounders, including age, gender, hypertension, diabetes, NIHSS on admission, and other variables with *P* < 0.20 when analyze by univariate analysis. The p values are considered significant at 0.05. Statistical analyses are performed using SPSS 16.0 (IBM, Armonk, NY).

**Table 2:** Clinical and subclinical characteristics of patients.

***Patient Characteristics***
*Age (IQR)*
*Gender*
*Initial SBP*, *mmHg*
*Initial DBP*, *mmHg*
*Initial NIHSS (IQR)*
*Disabling*
***Hospitalization Time window***
*< 4*,*5h*
*4*,*5–6 h*
*6–12 h*
*12–24 h*
***TOAST classification***
*Large-artery atherosclerosis*
*Cardioembolism*
*Small-vessel occlusion*
*Other determined etiology*
*Undetermined etiology*
***Large cerebral artery occlusion***
***Location of large vessel occlusion***
*ICA*
*M1*
*M2*
*BA*
*PCA*
*VA*
***Reperfusion treatment***
*rtPA*
*Alteplase 0*,*6mg/kg*
*Alteplase 0*,*9mg/kg*
*Thrombectomy*
***Risk of stroke***
*Hypertension*
*Atrial Fibrillation/Atrial Flutter*
*Diabetes*
*High blood cholesterol*
*Smoking*
*Alcohol abuse*
*Obese*
*Previous Ischemic stroke/TIA*
*Coronary artery disease/MI*
*Heart failure*
***Cerebral vascular imaging***
*Multifocal cerebral infarction*
*Corresponding intracranial artery stenosis > 50%*
*Corresponding extracranial artery stenosis > 50%*
***Internal treatment***
***Anticoagulation***
*Rivaroxaban*
*Apixaban*
*Dabigatran*
***Antiplatelet***
*Aspirin*
*Clopidogrel*
*Ticagrelor*
*Dual antiplatelet*

### Primary outcome

The primary result of this research is to determine the END population rate in the subgroup of minor stroke patients in our stroke centre.

### Secondary outcomes

The primary outcome provides quantitative information on patient outcomes, frequency and predictors for the END population in Vietnam. Furthermore, a statistical predictive model could be calculated to Identify END prognostic factors based on logistic regression analysis.

Table 2 shows clinical parameters, and treatment characteristics in patients with minor stroke in the study population, the group with END and the group without END. This table will determine the study’s primary goal, the END rate. At the same time, it also determines the different characteristics between the two groups with END and without END. In particular, intracranial artery stenosis is considered characteristic in Asian patients with ischemic stroke; we will determine the rate of intracranial artery stenosis and the relationship with END.

We then continued to discover the differences in clinical, paraclinical, and treatment characteristics between the END and non-END groups. Logistic regression analysis was performed to identify factors related to and prognostic of END to answer the second goal.

## Discussion

The significant contribution of the article is to determine the END rate and prognostic factors for END in a group of patients with minor stroke at a comprehensive stroke centre in Vietnam. From there, it helps guide clinical practice inappropriately approaching diagnosing and treating patients with minor strokes in the emergency room. The American Stroke Association recommends that patients with minor strokes with disabling neurological deficits be prescribed thrombolytic drugs, while groups without disabling neurological deficits will not be recommended for thrombolysis. Recently published research by Chen, et al. demonstrated that thrombolysis and antiplatelet drugs are effective in patients with minor strokes without neurological deficits—equal clinical benefits [16].

Patients with minor strokes with NIHSS ≤ 5 points are often excluded from mechanical thrombectomy studies. Recent studies have shown that mechanical thrombectomy in patients with minor stroke with low NIHSS scores in large artery occlusion might not demonstrate clinical benefit [17–19]. Research conducted across multiple centres and looking back at past cases has revealed that using endovascular mechanical thrombectomy does not provide any advantage over other treatment approaches for minor stroke patients with isolated M2 occlusion [20]. To reduce poor clinical outcomes and complications, early identification of risk factors and closely monitoring may help prevent complications and maximize the benefits of endovascular thrombectomy [21, 22]. Intracranial artery stenosis is more common in Asians than in Westerners. Is intracranial artery stenosis a risk factor for END? Our study will help to identify END risk factors and allow rational treatment choices for patients presenting within the reperfusion window (thrombolysis and mechanical thrombectomy).

In other research, in 2020, Sung SM, et al. demonstrated that Hemorrhagic transformation, initial NIHSS score, stenosis, or occlusion of the relevant artery were, respectively, prognostic factors for END in a group of minor strokes [12]. However, in Sung’s study, the criteria for minor stroke patients is NIHSS score ≤ 3. In 2021, Xiong, et al. found that patients with minor stroke had a rate of treatment outcomes, mortality, recurrent stroke, and myocardial infarction in two groups (NIHSS ≤ 3 points and NIHSS ≤ 5 points) are equivalent [23]. Therefore, an NIHSS score ≤ 5 is a more suitable criterion for a minor stroke [1, 3, 23]. This is also the first study on END (NIHSS ≥ 2) in patients with minor stroke (NIHSS ≤ 5) and including large artery occlusions or not different from N. Boulenoir only selected patients with large artery occlusions [15].

Our study determined the END rate in patients with minor stroke NIHSS ≤ 5 and prognostic factors for END. From there, it is proposed that patients at risk of END should be hospitalized early and treated with aggressive strategies such as reperfusion therapy. This study also can potentially extend to investigate further into risk factors to END and also early warning methods for stroke prevention in minor stroke patients.
